# Surgical *versus* Non-Surgical Management of Obstructive Sleep-disordered Breathing in Children: A Meta-analysis

**DOI:** 10.2174/1874306402014010047

**Published:** 2020-11-26

**Authors:** Alaa Ahmed Abd El Hamid, Anas Mohamed Askoura, Diaa Marzouk Abdel Hamed, Mohamed Shehata Taha, Mohamed Farouk Allam

**Affiliations:** 1 Department of Family Medicine, Faculty of Medicine, Ain Shams University, Cairo, Egypt

**Keywords:** OSDB, Children, Surgical, Meta-analysis, Systematic review, Quality of life

## Abstract

**Background:**

Obstructive sleep-disordered breathing (OSDB) is a term for several chronic conditions in which partial or complete cessation of breathing occurs many times throughout the night, resulting in fatigue or daytime sleepiness that interferes with a person’s functions and reduces the quality of life.

**Objective:**

Comparing the effectiveness of surgical *versus* non-surgical treatment of OSDB in children in clinical trials through a meta-analysis study.

**Patients and Methods:**

A number of available studies and abstracts concerning the surgical *versus* non-surgical treatment of OSDB in children were identified through a comprehensive search of electronic databases. Data were abstracted from every study in the form of a risk estimate and its 95% confidence interval.

**Results:**

The current study revealed that there was a statistically significant improvement in the surgically treated patients rather than non-surgically treated patients regarding the quality of life.

**Conclusion:**

The current meta-analysis reports a significant clinical improvement in the surgical (adenotonsillectomy) group as compared to the non-surgical group, in terms of disease specific quality of life, and healthcare utilization in spite of the availability of only one study.

## 1 INTRODUCTION

Obstructive Sleep-Disordered Breathing (OSDB) is a term for several chronic conditions in which partial or complete cessation of breathing occurs many times throughout the night, resulting in fatigue or daytime sleepiness that interferes with a person’s functions and reduces the quality of life [1].

OSDB can occur in both children and adults; it ranges in severity from simple snoring to Obstructive Sleep Apnea Syndrome (OSAS) [2].

Obstructive sleep apnea (OSA) is a common condition known to affect 1–4% of all children [3], the estimated prevalence of habitual snoring in children is 4.9 to 12.1% [4].

Infants often present with noisy breathing and disturbed nocturnal sleep, preschool-aged children with snoring and mouth breathing, and school-aged children present with behavioral and dental problems [2].

The clinical presentation of a child with OSA syndrome is broad-based and requires increased awareness by the primary care physician, as it has a variety of presenting symptoms other than snoring, and therefore, clinicians must be alert to these symptoms [5].

Pediatric obstructive sleep apnea is a multifactorial condition; with anatomical and neuromuscular components etiologies including anatomical abnormalities, obesity, hypo- sthyroidism, neurologic disorders, and Down syndrome [6].

In children, hypertrophy of the tonsils and adenoid tissue is assumed to be the most common cause of OSDB; it causes narrowing of the airway, resulting in a problem during sleep when the muscles of the pharynx relax, leading to partial or complete obstruction of the airway [7].

Complications range from neurocognitive effects, such as hyperactivity and poor school performance and growth restriction, to cardiovascular and pulmonary hypertension [6]; untreated pulmonary hypertension in children can lead to right heart failure with decreased cardiac output and may contribute to increased morbidity and mortality [8].

The diagnosis of SRBD based on history, physical findings, and polysomnography is considered the most comprehensive investigation for diagnosing obstructive sleep apnea syndrome [9]. Other diagnostic studies may be needed to evaluate complications of obstructive sleep apnea or to assess the underlying conditions [10].

The treatment options for SRBD in children can be divided into four categories, a surgical approach with adenotonsillectomy, tracheostomy, or other maxillofacial surgery, a mechanical approach with positive airway pressure, a conservative approach with observation, weight loss (in the obese child), and finally, a medical approach using oxygen or some type of medication [11].

Guidelines from both the American Academy of Otolaryngology–Head and Neck Surgery and the American Academy of Pediatrics recommend adenotonsillectomy (T&A) as the first-line treatment [9].

A recent meta-analysis (2009) indicates a 34% to 40% likelihood of persistent OSA following T&A. The most common complication is post-operative bleeding, which may occur in up to 5% of children [12].

CPAP therapy is delivered by using an electronic device that delivers air at positive pressure via a nasal mask, leading to mechanical stenting of the airway and improved functional residual capacity in the lungs [9]. Nasal corticosteroids exert lympholytic action to treat inflammation and upper airway edema, nasal fluticasone for 6 weeks significantly decreased both adenoid size and AHI [13]. Leukotriene levels have also been shown to be elevated in the adenotonsillar tissue of children with OSAS compared to those with tonsillitis [14]. Despite recent clinical guideline development, the best pathway of care for children with symptoms of Obstructive Sleep-Disordered Breathing (OSDB) is still debated [15].

The objective of this meta-analysis was to compare the effectiveness of surgical *versus* non-surgical treatment of obstructive sleep-disordered breathing in children.

## 2 MATERIALS AND METHODS

### 2.1 Literature Review

Available studies and abstracts concerning the surgical *versus* non-surgical treatment of Obstructive sleep-disordered breathing in children were identified through a comprehensive search of electronic databases, using a combination of the following keywords: OSDB, Children, surgical, meta-analysis, quality of life.

The following outcomes were assessed in the study: disease-specific quality of life, health care utilization, adverse events, and complications of treatment.

Studies concerned with the treatment of Obstructive sleep-disordered breathing in children, studies conducted on children aged 2 to 16 years, studies conducted on human subjects, published in Arabic, English, French and Spanish languages were included.

Opinionated studies, RCTs that included children with central SDB (*e.g.*, SDB related to neurological conditions or brain injury), studies on apnea of prematurity, and studies conducted on animals, studies published in other languages “not in Arabic, English, French or Spanish”, and studies conducted on adults were excluded.

### 2.2 Data Extraction

Seven published studies were identified on the basis of the inclusion criteria [16-22]. A copy of each identified study was obtained, and relevant data were abstracted by the first author (A.A.A.) for a quantitative overview. The type of risk estimate (*i.e.*, relative risk or odd's ratio) and the country where the study was carried out were also ascertained. In case of discrepancies or when the information presented in a study was unclear, abstraction by a second reviewer (M.F.A.) was sought to resolve the discrepancy.

### 2.3 Statistical Methods for Meta-Analysis

Data were abstracted from every study in the form of a risk estimate and its 95% confidence interval.

Pooled estimates of relative risks were obtained by weighing each study by the inverse variance of the effect measure on a logarithmic scale. This approach to pool the results assumes that the study populations being compared are similar and hence corresponds to a fixed effect analysis. The validity of pooling the relative risks was tested (test of homogeneity) using the chi-square test. A violation of this test suggests that the studies being pooled differ from one another. In the presence of significant heterogeneity of the effect measure among studies being compared, a random effect analysis was performed that was based on the method described by DerSimonian and Laird. The random effect analysis accounts for the interstudy variation. Because the test of homogeneity has low power, the figures of the random effect analysis were reported even with the absence of significant heterogeneity.

Statistical analysis was done using the STATA Statistical Software, release 14.0 (StataCorp. 2015) (Table **1** and **2**).

## 3 RESULTS

The seven studies, which had been identified, were carried out between 2004 and 2014. Using appropriate statistical methods, comparisons were made as regards disease specific quality of life, health care utilization, adverse events, and complications of treatment in both groups. Surgery in localized studies was indicated in children with adenotonsillar growth.

### 3.1 Pooled Results that Evaluated Disease-Specific Quality of Life

**Fig. (1):**
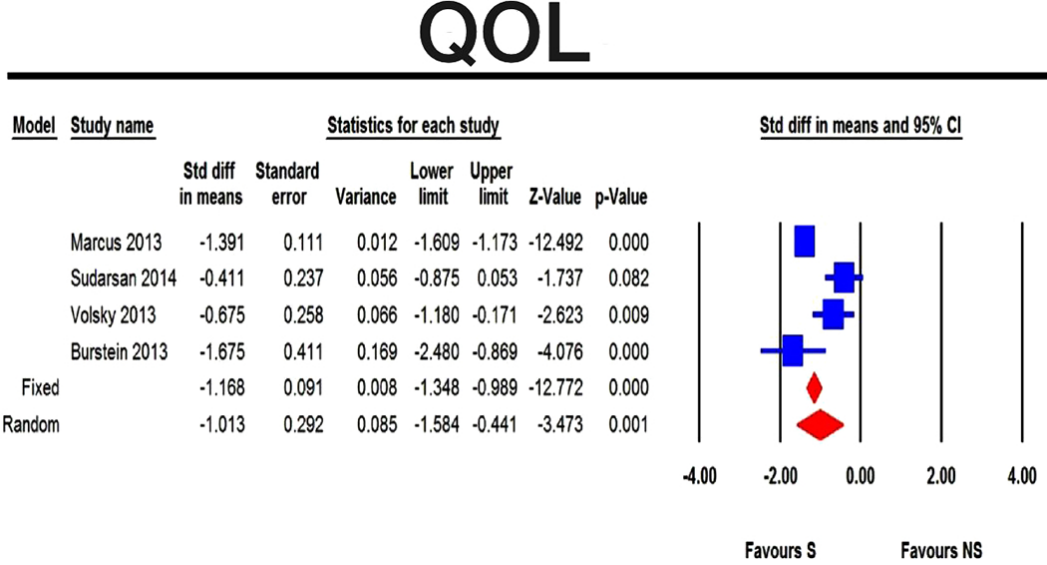
Forest plot showing the difference between surgical (S) and non-surgical (NS) management obstructive sleep-disordered breathing in children as regards the quality of life..

 The four pooled studies that evaluated the change in disease specific quality of life among children with obstructive sleep disordered breathing showed clear clinical improvement in the surgical group compared with the non-surgical group (p-value <0.001) (Fig. **1**). Comparison with CPAP was not shown alone, but it was contained in the non-surgical panel of treatment. None of the localized studies compared surgery with CPAP regarding QOL.

### 3.2 Pooled Results that Evaluated Healthcare Utilization

 Only one study measured Healthcare utilization, which states that adenotonsillectomy significantly reduces health care utilization in children with obstructive sleep apnea syndrome [20]. Untreated children with moderate and severe obstructive sleep apnea syndrome will continue to consume high levels of health care resources.

### 3.3 Pooled Results that Evaluated Adverse Events

**Fig. (2):**
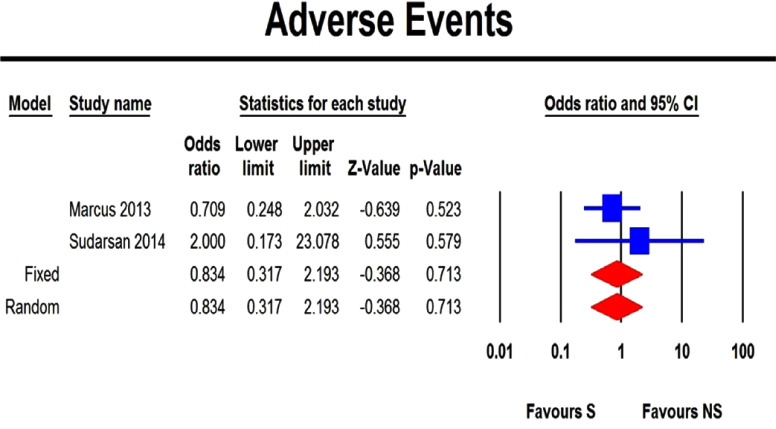
Forest plot showing the difference between surgical (S) and non-surgical (NS) management obstructive sleep-disordered breathing in children as regards the risk for adverse events..

The two pooled studies that evaluated the adverse events that occurred among children with obstructive sleep disordered breathing treated either by surgical or non-surgical treatment showed no statistically significant difference in adverse events of surgery compared with the non-surgical management (p-value 0.713) (Fig. **2**).

## 4 DISCUSSION

OSDB results from obstruction or dynamic collapse of upper airway soft tissues during sleep, which can manifest as snoring, hypopnea, apnea, and restless sleep ***^(22)^.***

Treatment involves surgical treatment (adenotonsi- llectomy) and non-surgical treatment (weight loss, CPAP, intranasal steroids, antileukotriens) [7].

This meta-analysis included studies regarding the effectiveness of tonsillectomy compared to non-surgical treatment of OSDB in children. The included studies are seven in number with a total of 849 patients arranged in two groups (surgically treated group = 429 and non-surgically treated group = 420) and all studies were published between the year 2004 and 2014.

Before reaching conclusions based on the present results, it is necessary to consider several potential objections to our procedures. Methodological concerns include limitations in the quality of the primary data, as the usefulness of a quantitative review largely depends on the quality of the studies used. It is of no doubt that combining randomized controlled trials provides more evidence than combining data from observational studies. However, a comprehensive search has identified seven studies with substantially different numbers of cases (32, 56, 59, 64, 124, 270 and 464), an insufficient number to obtain reliable results. Also, studies were of different designs, population, length of follow up, and outcome measures were heterogeneous.

### 4.1 Pooled Results that Evaluated Disease-specific Quality of Life

The four pooled studies that evaluated the change in disease-specific quality of life among children with obstructive sleep disordered breathing [16-18, 21] showed clear clinical improvement in the surgical group compared with the non-surgical group (p-value <0.001). The results coincide with that of a recent meta-analysis of Venekamp [7], where they pooled only three studies.

The heterogeneity test between the pooled studies show a significant difference, which indicates interstudy variation and pooling of this heterogeneous set of studies add more useful information; moreover, the fixed effect model compared with the random effect model showed no difference.

The difference between the results of the four studies could be attributed to the population of the studies. Marcus [16] conducted a study on children aged 2-5 years with mild to moderate OSA while, Sudarsans’ [17] population was syndromic (children with down syndrome and mucopolysaccharidosis) aged 6 to 12 years patients, Volsky [18] conducted a study on children aged 3 to 16 years with mild obstructive sleep apnea and Brustein [21] studied obese children aged one to 12 years. Moreover, Marcus, Sudarsan, Volsky used OSA-18 questionnaire to evaluate the disease-specific quality of life, while Brustein used the clinical assessment score-15 (CAS-15) for the same outcome assessment.

The tolerance index for the quality of life equals 7; this means that 7 unpublished studies with negative results are needed to convert this meta-analysis to the negative side.

### 4.2 Pooled Results that Evaluated Healthcare Utilization

Only one study measures Healthcare utilization [20], which states that adenotonsillectomy significantly reduces health care utilization in children with OSA syndrome. Untreated children with moderate and severe obstructive sleep apnea syndrome will continue to consume high levels of health care resources. This study was conducted at clalit health care services in the south region of Israel, mainly on children with severe OSA syndrome.

### 4.3 Pooled Results that Evaluated Adverse Events

The two pooled studies that evaluated the adverse events that occurred among children with obstructive sleep disordered breathing treated either by surgical or non-surgical treatment [16, 17] showed no statistically significant difference in adverse events of surgery compared with the non-surgical management (p-value 0.713), and this ensures that the risk of surgery does not carry more danger than non-surgical treatment statistically.

The heterogeneity test between the pooled studies showed non-significant difference as regards the adverse events and the fixed effect model, compared with the random effect model, showed no difference in both.

## CONCLUSION

In conclusion, the current meta-analysis reports a significant clinical improvement in the surgical (adenotonsillectomy) group as compared with the non-surgical group, in terms of disease-specific quality of life, and healthcare utilization in spite of the availability of only one study.

## Figures and Tables

**Table 1:** Meta-analysis for the difference between surgical and non-surgical management of obstructive sleep-disordered breathing in children as regards the quality of life.

** -**	** -**			** 95% CI**		** -**	** -**
** Study **	** SMD**	** SE**	** Variance**	** Lower limit**	** Upper limit**	** Z-value**	** P-value**
** Marcus *et al*. [16]**	-1.391	0.111	0.012	-1.609	-1.173	-12.492	** <0.001**
** Sudarsan *et al*. [17]**	-0.411	0.237	0.056	-0.875	0.053	-1.737	0.082
** Volsky *et al*. [18]**	-0.675	0.258	0.066	-1.180	-0.171	-2.623	** 0.009**
** Burstein *et al*. [21]**	-1.675	0.411	0.169	-2.480	-0.869	-4.076	** <0.001**
* FEM*	* -1.168*	* 0.091*	* 0.008*	* -1.348*	* -0.989*	* -12.772*	*** <0.001***
* REM*	* -1.013*	* 0.292*	* 0.085*	* -1.584*	* -0.441*	* -3.473*	*** 0.001***
** Heterogeneity**							
** Q-value**	** df (Q)**	** P-value**	** I-squared**	** -**	** -**	** -**	** -**
19.437	3.000	** <0.001**	84.566	-	-	-	-

95% CI = 95% confidence interval, SMD, standardized mean difference; SE = standard error, FEM = fixed effects model, REM = random effects model, df (Q) = degree of freedom.

**Table 2:** Meta-analysis for the difference between surgical and non-surgical management of obstructive sleep-disordered breathing in children as regards the risk for adverse events.

-	-	**95% CI**		-	-
**Study**	**Odds ratio**	**Lower limit**	**Upper limit**	**Z-value**	**P-value**
**Marcus *et al*. [16]**	0.709	0.248	2.032	-0.639	0.523
***Sudarsan *et al*.* [17]**	2.000	0.173	23.078	0.555	0.579
*FEM*	*0.834*	*0.317*	*2.193*	*-0.368*	*0.713*
*REM*	*0.834*	*0.317*	*2.193*	*-0.368*	*0.713*
**Heterogeneity**					
**Q-value**	**Df (Q)**	**P-value**	**I-squared**	-	-
0.582	1.000	0.445	0.000	-	-

95% CI = 95% confidence interval, FEM = fixed effects model, REM = random effects model, df (Q) = degree of freedom.
